# Electrical Stimulation of Injected Muscles to Boost Botulinum Toxin Effect on Spasticity: Rationale, Systematic Review and State of the Art

**DOI:** 10.3390/toxins13050303

**Published:** 2021-04-23

**Authors:** Alessandro Picelli, Mirko Filippetti, Giorgio Sandrini, Cristina Tassorelli, Roberto De Icco, Nicola Smania, Stefano Tamburin

**Affiliations:** 1 Department of Neurosciences, Biomedicine and Movement Sciences, University of Verona, 37100 Verona, Italy; alessandro.picelli@univr.it (A.P.); mirko.filippetti@univr.it (M.F.); nicola.smania@univr.it (N.S.); 2 Department of Brain and Behavioral Sciences, University of Pavia, 27100 Pavia, Italy; giorgio.sandrini@unipv.it (G.S.); cristina.tassorelli@unipv.it (C.T.); roberto.deicco@mondino.it (R.D.I.); 3 Headache Science & Neurorehabilitation Center, IRCCS Mondino Foundation, 27100 Pavia, Italy

**Keywords:** botulinum toxins, electrical stimulation, muscle spasticity, physical therapy modalities, rehabilitation

## Abstract

Botulinum toxin type A (BoNT-A) represents a first-line treatment for spasticity, a common disabling consequence of many neurological diseases. Electrical stimulation of motor nerve endings has been reported to boost the effect of BoNT-A. To date, a wide range of stimulation protocols has been proposed in the literature. We conducted a systematic review of current literature on the protocols of electrical stimulation to boost the effect of BoNT-A injection in patients with spasticity. A systematic search using the MeSH terms “electric stimulation”, “muscle spasticity” and “botulinum toxins” and strings “electric stimulation [mh] OR electrical stimulation AND muscle spasticity [mh] OR spasticity AND botulinum toxins [mh] OR botulinum toxin type A” was conducted on PubMed, Scopus, PEDro and Cochrane library electronic databases. Full-text articles written in English and published from database inception to March 2021 were included. Data on patient characteristics, electrical stimulation protocols and outcome measures were collected. This systematic review provides a complete overview of current literature on the role of electrical stimulation to boost the effect of BoNT-A injection for spasticity, together with a critical discussion on its rationale based on the neurobiology of BoNT-A uptake.

## 1. Introduction

Botulinum neurotoxin type A (BoNT-A) is a single-chain polypeptide of about 150,000 Da molecular weight produced by *Clostridium botulinum* [1]. BoNT-A selectively inhibits the release of acetylcholine (Ach) at peripheral cholinergic nerve endings, causing chemical denervation due to the cleavage of SNAP-25 protein, a part of the SNARE complex, which is required for vesicle docking and, consequently, Ach release [2]. The action of BoNT-A at nerve endings consists of the following four steps: (1) binding, (2) internalization, (3) translocation and (4) proteolysis [3]. BoNT-A is composed of a heavy chain and a light chain linked together by a single disulfide bond, with the C-terminal of the heavy chain mainly responsible for binding; its N-terminal is involved in the membrane translocation process, while the light chain exerts the intracellular catalytic (metalloprotease) activity [1,2,3].

BoNT-A binds with high affinity to the presynaptic cell membrane of skeletal cholinergic nerve terminals. This peculiar tropism and catalytic activity, which leads to neuromuscular transmission transient blockade, are the basis of the therapeutic use of BoNT-A in patients with neurological conditions associated with overactive muscle conditions [4]. In particular, BoNT-A represents a first-line treatment for the management of spasticity [5,6], which may occur as a consequence of several neurological conditions affecting adults and children, such as stroke, multiple sclerosis, traumatic brain injury, spinal cord injury, tumors, neurodegenerative disorders and cerebral palsy [6,7,8]. The term spasticity refers to a disordered sensorimotor control, presenting as intermittent or sustained involuntary involvement of muscles [7]. In addition to BoNT-A, other treatment options for managing spasticity are oral medications, chemodenervation with phenol or alcohol, intrathecal baclofen, rehabilitation procedures, physical modalities, and surgical interventions [9,10].

### 1.1. Issues in the Management of Spasticity with Botulinum Toxin and Rationale to Boost Its Effect

To date, three brands of BoNT-A are approved for treating spasticity in clinical practice: abobotulinumtoxinA (Dysport, Ipsen, Boulogne-Billancourt, France), incobotulinumtoxinA (Xeomin, Merz, Frankfurt am Main, Germany) and onabotulinumtoxinA (Botox, Allergan, Irvine, CA, USA) [5,11]. Their efficacy and safety have been demonstrated for labeled doses, which may vary across countries according to their own regulations [5]. In real-world practice, to achieve appropriate clinical and neurorehabilitative goals, the treatment of multifocal spasticity may require doses of BoNT-A that are higher than the recommended regimen [12]. Thus, depending on the clinical presentation of spasticity in some patients, the cumulative and/or per muscle dose of BoNT-A may be higher than that recommended by the product label [13,14]. Adverse events and antibody development must be considered as potential risks of high-dose BoNT-A therapy [12]. To date, the safety of BoNT-A injection regimens at higher than label-recommended doses is supported by growing evidence [12,15,16,17,18], although the theoretical risk of local side effects due to the spreading of BoNT-A to the adjacent muscles cannot be ruled out [19]. Furthermore, the data regarding the safety of prolonged use of high BoNT-A doses are limited [12,15,20]. In this frame, it is worth noting that the development of neutralizing antibodies seems to be favored by long-duration therapy with high doses and short intervals between treatment cycle injections, although neutralizing antibodies have been reported in only 1% of subjects treated with BoNT-A for limb spasticity, without significant difference between brands [21]. Lastly, it is noteworthy that high-dose BoNT-A treatments may be more expensive than on-label ones [19,22].

Therapeutic strategies to boost the effect of BoNT-A thus seem useful to overcome the issues described above and may lead to some advantages, such as the injection of lower BoNT-A dosages, fewer side effects and lower costs.

### 1.2. Electrical Stimulation as Booster for Botulinum Toxin Effect

To date, several rehabilitation procedures have been proposed as adjuvant treatments associated with BoNT-A injection for the management of spasticity, including muscle stretching; taping; casting; splinting; orthoses; and physical modalities such as extracorporeal shock wave therapy, therapeutic ultrasound, vibration therapy, electrical stimulation, and transcutaneous electrical nerve stimulation [23,24].

Rat diaphragm preparation studies conducted in the 1960s suggested a reduction in latency of the onset of BoNT-A paralytic effect after electrical stimulation of motor nerve endings [25]. Specifically, electrical stimulation was shown to enhance the neuromuscular blockade effect of BoNT-A by increasing and accelerating the toxin uptake at the motor nerve terminals in animal models [25,26]. In particular, electrical stimulation may speed up the binding/internalization and translocation of BoNT-A, which have half-times of approximately 12 and 5 min, respectively [26]. Furthermore, studies on cultured neuron models showed not only a quick (i.e., occurring a few minutes after toxin exposure) internalization of BoNT-A due to endocytosis mediated by high-affinity receptors but also a protective effect of pH neutralizing substances against BoNT-A intoxication less than 40 min after toxin exposure [1,27].

In the same line, evidence in humans without spasticity suggested an important role of the injected muscle activity in the clinical response to BoNT-A [28]. In 1995, Hesse and colleagues were the first to investigate the possibility of enhancing the effect of BoNT-A by means of electrical stimulation in patients with stroke and spasticity [29]. Since then, several electrical stimulation protocols have been proposed to boost the effect of BoNT-A in patients with spasticity. Despite the encouraging evidence reported by many studies, there is no agreement on several parameters, such as the time of administration after BoNT-A injection, the stimulation frequency, and the duration of stimulation sessions [19].

To offer a critical update on the state of the art in this still-controversial topic, we have systematically reviewed the literature on the use of electrical stimulation to boost the effect of BoNT-A on spastic muscles.

## 2. Results

We included a total of sixteen articles, whose findings are described below.

### 2.1. Adult Patients

Nine studies (randomized controlled trial: *n* = 8, case-control study: *n* = 1; chronic stroke patients: *n* = 8, lower-limb spastic paraparesis: *n* = 1) were included [29,30,31,32,33,34,35,36,37]. Four studies dealt with upper limb spasticity with the following muscles as injection targets: biceps brachii, flexor carpi radialis, flexor carpi ulnaris, flexor digitorum superficialis, flexor digitorum profundis and abductor digiti minimi [32,35,36,37]. Five studies were conducted on patients with lower limb spasticity and considered the following muscles as injection targets: gastrocnemius, soleus, tibialis posterior and extensor digitorum brevis [29,30,31,33,34]. AbobotulinumtoxinA was used in four studies (upper limb: *n* = 1, lower limb: *n* = 3) [29,33,34,35]. OnabotulinumtoxinA was used in five studies (upper limb: *n* = 3, lower limb: *n* = 1; both upper and lower limb: *n* = 1) [30,31,32,36,37]. Protocols for electrical stimulation utilized 30–60-min sessions. There was great variability in terms of session frequency (range: 1–6 times/day), total number (range: 1–18) and duration of the stimulation cycles (range: 1–6 days). The electrical stimulation of the injected muscles was started immediately after BoNT-A injection in three studies [30,36,37] and was started the day after injection in three studies [32,34,35]; three studies generically reported that BoNT-A injection was followed by electrical stimulation [29,31,33]. One study used sham stimulation (i.e., electrodes positioned but no stimulation given) [32]. Studies differed in terms of stimulation protocol parameters and the combination of electrical stimulation with other post-injection strategies to improve the effect of BoNT-A. Two studies reported adverse events after treatment. Table 1 includes detailed information on the treatment protocols and outcomes.

### 2.2. Pediatric Patients

We included seven studies (randomized controlled trial: *n* = 5, case-control study: *n* = 2) on children with spastic cerebral palsy [38,39,40,41,42,43,44]. Injection targets were hip adductors, hamstrings, and calf (i.e., gastrocnemius, soleus and tibialis posterior) muscles as well as upper limb muscles (i.e., biceps brachii, brachioradialis, pronator teres, flexor carpi radialis and ulnaris, adductor pollicis and flexor pollicis brevis). OnabotulinumtoxinA was used in all studies. Except for two studies, which used electrical stimulation sessions of 15 and 20 min/session respectively [43,44], the remaining electrical stimulation protocols utilized 30-min sessions, but there was great variability in terms of treatment session frequency (range: 6 times/day to 1 time/week), total number of sessions (range: 4–60) and duration of treatment cycles (range: 3 days to 12 weeks). Only two studies reported that electrical stimulation of the injected muscles occurred on the same day as BoNT-A injection (the time between injection and electrical stimulation was specified by one paper) [38,44]. Conversely, the other studies generically reported that BoNT-A injection was followed by electrical stimulation, except for one study that reported the start of electrical stimulation three weeks after injection [43]. One study used sham stimulation, but details of sham stimulation were not reported [42]. Studies differed in terms of stimulation protocol parameters and the combination of electrical stimulation with other post-injection strategies to improve the effect of BoNT-A. None of the studies reported adverse events after treatment. Table 2 includes detailed information on the treatment protocols and outcomes.

## 3. Discussion

Therapeutic electrical stimulation is widely used in neurorehabilitation for strengthening muscles and improving motor recovery [45]. Furthermore, electrical stimulation has been reported to reduce spasticity “per se”, although the mechanism remains uncertain, and the clinical evidence is equivocal [46].

Electrical stimulation has been frequently used as an adjunct treatment to boost the effect of BoNT-A based on the idea that it might increase its uptake in muscles affected by spasticity both in adults and children [23]. When considering that BoNT-A uptake takes place a few minutes after BoNT-A injection [1,25,26,27,28], electrical stimulation of the injected muscle should be delivered early after BoNT-A injection, and the duration and number of stimulation sessions should be based on the toxin uptake time course [1,27].

The output of this systematic analysis of the literature shows that a wide range of electrical stimulation protocols have been proposed to boost the effect of BoNT-A in spasticity [29,30,31,32,33,34,35,36,37,38,39,40,41,42], but only a few were designed considering the timing of toxin uptake. In particular, only four reports clearly specified that electrical stimulation was delivered immediately after BoNT-A injection (i.e., during toxin uptake) [30,36,37,43]. Three studies reported that electrical stimulation started the day after BoNT-A injection (i.e., at a time when toxin uptake is unlikely to be still in progress) [32,34,35]. One study specified that electrical stimulation started three weeks after BoNT-A injection [44]. Notably, the remaining eight studies did not specify the exact stimulation timing after BoNT-A treatment [29,31,33,38,39,40,41,42], even if it is likely that electrical stimulation was administered early after BoNT-A injection in most of them.

The duration of each stimulation session is in line with the timing of BoNT-A uptake (30–60 min) in all studies, except for two reports that applied stimulation for 15 and 20 min/session, respectively [43,44]. However, several stimulation sessions (i.e., up to 60) over hours/days, in treatment cycles lasting up to 12 weeks, were applied in many studies. The timing of BoNT-A uptake is relatively short and not prolonged or repeated over time. Thus, the application of multiple stimulation sessions applied on the same day and/or over long periods of time does not seem to have a robust neurobiological foundation [1,27].

Interestingly, despite the large variability in stimulation protocols and their poor adherence to the BoNT-A uptake timing, most included studies reported significant effects on clinical and neurophysiological outcomes in response to electrical stimulation in combination with BoNT-A injection in comparison to control conditions [29,30,31,32,33,34,35,36,37,38,39,40,41,42,44]. We speculate that this boosting effect of electrical stimulation on BoNT-A treatment may be due to the first stimulation session, presumably delivered early after toxin administration in most studies (see above). The benefits achieved in patients managed with delayed administration of electrical stimulation or longer stimulation protocols may be related to the unspecific effect of electrical stimulation to reduce spasticity “per se” rather than to a specific boosting effect of electrical stimulation on BoNT-A injection. Furthermore, the effect of other adjuvant treatments (e.g., stretching, splinting, physical therapy) combined with BoNT-A injection and electrical stimulation might have accounted for the findings reported in some of the included studies.

Of note, only two papers reported some mild adverse events after electrical stimulation following BoNT-A injection [29,32], supporting the overall safety of electrical stimulation protocols for adult and pediatric patients with spasticity.

Our observations are in line with a previous systematic review by Intiso and colleagues, which concluded that electrical stimulation may boost BoNT-A injection action and reduce adult spasticity [19]. Nevertheless, due to the variability in protocols and the paucity of high-quality trials, the authors did not recommend the combination of BoNT-A injection and electrical stimulation in clinical practice [19]. Our review is methodologically different, because we focused on the use of neuromuscular stimulation of the injected muscles to boost the effect of BoNT-A on spasticity in adults and children, and we included all study types. On the other hand, Intiso and colleagues included only randomized controlled trials involving adult patients and considered all the types of stimulation (i.e., neuromuscular stimulation, transcutaneous electrical nerve stimulation and functional electrical stimulation), further increasing the variability in protocols and reducing the possibility of strong conclusions [19]. Another systematic review by Mathevon and colleagues did not recommend electrical stimulation as an adjunct therapy to BoNT-A injection [47]. In our view, this conclusion is very limited considering that the authors included only four studies, i.e., two on functional electrical stimulation and two on neuromuscular stimulation, in children with spastic cerebral palsy [47].

## 4. Conclusions

Electrical stimulation may boost the effect of BoNT-A, as suggested by most studies evaluated in this review. The hypothesized mechanism of action of electrical stimulation is the enhancement of the BoNT-A uptake process at the presynaptic cholinergic nerve terminals [1,25,26,28]. Thus, from a neurobiological perspective, electrical stimulation should be applied to the injected muscles during the BoNT-A uptake process, which occurs rapidly after injection, to exert a boosting effect in patients with spasticity [1,25,27]. In this line, from a practical point of view, a single electrical stimulation session performed on the same day of BoNT-A injection would be preferable, in that it would facilitate the access to care for patients by reducing the number of visits to hospitals and rehabilitation centers, as well as reducing the burden of caregivers [48]. Unfortunately, most of the studies evaluated in this review adopted longer stimulation protocols; thus, it is not possible to verify whether a single stimulation session lasting 30–60 min applied on the injected muscles immediately after BoNT-A administration might be sufficient to boost the effect of BoNT-A in the treatment of spasticity [26,27]. Furthermore, a “per se” antispastic effect of electrical stimulation should be considered [19,46]. From this perspective, the eventual contribution of electrical stimulation itself in alleviating spasticity represents an under-investigated issue, as well as a limit for the relevance of any potentially specific effects on BoNT-A injection for spasticity. Thus, there is a need to clearly demonstrate that the effect of electrical stimulation combined with BoNT-A is more than additive.

Despite the amount of literature about the use of electrical stimulation to boost the effect of BoNT-A on spasticity, some questions remain open. Do all the injected muscles have to be stimulated to improve a specific pattern of spasticity? Which part of the muscle is the one best suited for stimulation? What is the difference in effect after electrical stimulation of the injected muscles compared to electrical stimulation of the supplying nerve? What is the most appropriate sham stimulation procedure for the control group?

Some additional issues come from neurophysiological considerations. First, the sustained spontaneous motor unit activity of spastic muscle causes high intrinsic Ach exocytosis and recycling activity favorable to BoNT-A uptake. In such a context, it is unclear whether the stimulation frequency applied to injected muscle may add to the baseline motor nerve discharge frequency of spastic muscles. Future studies incorporating monitoring of the discharge frequency in motor nerves will help to answer this question [49]. Second, the link between muscle stimulation and the activity of motor neuron terminals, where the exo/endocytosis activity responsible for BoNT-A uptake takes place, is unclear. Electrical stimulation of the injected muscle at the frequency used in the studies we have reviewed may induce muscle contraction but may not be able to stimulate motor nerves or motor-nerve terminals. Third, muscle contraction secondary to electrical stimulation does not seem to alter the diffusion pattern or bolus distribution of BoNT [50]. Fourth, BoNT-induced muscle atrophy might paradoxically reduce the effect of Ach release inhibition secondary to BoNT injection. From this perspective, prolonged electrical stimulation of the muscle could prevent muscle fiber atrophy, thus increasing the effect of BoNT. Fifth, muscle stimulation may retroact on the presynaptic endings and interfere with proprioceptive information and sensory–motor integration. Because of these lines of reasoning, electrical stimulation during several days might also modulate the effect of BoNT-A injection in spastic muscles.

Because of the wide range of protocols proposed in the literature and their limitations (e.g., the stimulation strength has not been reported in many studies), together with the great variability in the outcome measures, and considering that the majority of studies included in this review have a small sample size, with a consequent lack of power, we encourage the conduction of further adequately powered multicenter randomized controlled trials to further support the role of electrical stimulation in improving the effect of BoNT-A on spasticity and define the most appropriate treatment schedule. In particular, we are not aware of any relevant in vivo study that explored the most appropriate dose and/or the dose–response relationship of electrical stimulation. These issues should be also addressed by future studies.

## 5. Materials and Methods

A systematic review using the MeSH terms “botulinum toxins”, “electric stimulation”, “muscle spasticity”, “physical therapy modalities” and “rehabilitation” and strings “electric stimulation [mh] OR electrical stimulation AND muscle spasticity [mh] OR spasticity AND botulinum toxins [mh] OR botulinum toxin type A” was conducted on PubMed, Scopus, PEDro and Cochrane Library electronic databases. The results were limited to original articles focusing on the use of electrical stimulation of injected muscles to boost the effect of BoNT-A therapy in adult patients and children suffering from spasticity. Thus, we excluded papers on animal models and humans without spasticity, as well as studies about the use of electrical stimulation for spasticity not associated with BoNT-A injection (e.g., functional electrical stimulation). Full-text articles written in English and published from database inception to March 2021 were included. Data on patient characteristics, treatment protocols and outcome measures were collected. Quality was assessed with the PEDro score.

## Figures and Tables

**Table 1:** Studies on adult patients.

Article	Summary
Hesse et al., 1995 [29]	*Design*: randomized controlled trial *Sample*: 11 chronic stroke patients affected by lower limb spasticity *Groups*: BoNT-A vs. BoNT-A + ES *BoNT-A injected*: abobotulinumtoxinA *Injected muscle(s)*: GM, GL, soleus, TP *Total BoNT-A dose injected*: up to 2000 U *Stimulation protocol*: 30-min sessions delivered 6 times/day for 3 consecutive days after injection *Stimulation parameters*: 20 Hz, 0.2 ms, 50–90 mA *Main findings*: BoNT-A + ES led to greater improvement in muscle tone, gait speed, stride length, stance, and swing-symmetry *Side effects reported*: one patient suffered from bladder paresis *PEDro score*: 4
Hesse et al., 1998 [35]	*Design*: randomized controlled trial *Sample*: 24 chronic stroke patients affected by upper limb spasticity *Groups*: BoNT-A + ES vs. BoNT-A vs. placebo + ES vs. placebo *BoNT-A injected*: abobotulinumtoxinA *Injected muscle(s)*: BB, Br, FCR, FCU, FDS, FDP *Total BoNT-A dose injected*: 1000 U *Stimulation protocol*: 30-min sessions delivered 3 times/day for 3 consecutive days after injection *Stimulation parameters*: 20 Hz, 0.2 ms, 50–90 mA *Main findings*: patients treated with BoNT-A + ES showed the greatest reduction in spasticity and facilitation of hand hygiene *Side effects reported*: none *PEDro score*: 5
Carda et al., 2005 [33]	*Design*: case-control study *Sample*: 65 chronic stroke patients affected by upper limb spasticity *Groups*: BoNT-A + taping vs. BoNT-A + ES + splinting *BoNT-A injected*: onabotulinumtoxinA *Injected muscle(s)*: FCR, FCU, FDS, FDP *Total BoNT-A dose injected*: up to 372 U (mean) *Stimulation protocol*: 60-min session, 1 time/day, 5 consecutive days *Stimulation parameters*: 50 Hz, 0.3 ms *Main findings*: patients treated with BoNT-A + taping achieved a greater reduction in spasticity with less time required for the treatment *Side effects reported*: none *PEDro score*: not applicable (estimated as 4)
Frasson et al., 2005 [34]	*Design*: randomized controlled trial *Sample*: 12 adult patients affected by spastic paraparesis due to multiple sclerosis, Strümpell–Lorrain disease, cerebral palsy and spinal cord injury *Groups*: BoNT-A + low-frequency ES vs. BoNT-A + high-frequency ES *BoNT-A injected*: abobotulinumtoxinA *Injected muscle(s)*: extensor digitorum brevis *Total BoNT-A dose injected*: 50 U *Stimulation protocol*: 30-min session, 1 time/day, 5 consecutive days *Stimulation parameters*: 4/25 Hz, 0.2 ms *Main findings*: low-frequency stimulation increased the effect of BoNT-A and induced a rapid and persistent improvement in spasticity *Side effects reported*: none *PEDro score*: 4
Bayram et al., 2006 [32]	*Design*: randomized controlled trial *Sample*: 11 chronic stroke patients affected by lower limb spasticity *Groups*: BoNT-A + ES vs. BoNT-A + sham ES *BoNT-A injected*: onabotulinumtoxinA *Injected muscle(s)*: GM, GL, soleus, TP *Total BoNT-A dose injected*: up to 400 U *Stimulation protocol*: 30-min sessions delivered 6 times/day for 3 consecutive days *Stimulation parameters*: 20 Hz, 0.2 ms, 50–90 mA *Main findings*: no significant between-group difference *Side effects*: pain at injection site (*n* = 4), muscle weakness (*n* = 2) *PEDro score*: 5
Baricich et al., 2008 [30]	*Design*: randomized controlled trial *Sample*: 23 chronic stroke patients affected by lower limb spasticity *Groups*: BoNT-A + ES + stretching vs. BoNT-A + taping vs. BoNT-A + stretching *BoNT-A injected*: abobotulinumtoxinA *Injected muscle(s)*: GM, GL *Total BoNT-A dose injected*: up to 500 U *Stimulation protocol*: 30-min sessions delivered 2 times/day for 5 consecutive days *Stimulation parameters*: 5 Hz, intensity adjusted according to the tolerance of patients *Main findings*: ES yielded greater reduction in spasticity (short-term follow-up); taping and ES lead to better improvement in all outcomes (long-term follow-up) *Side effects reported*: none *PEDro score*: 6
Picelli et al., 2011 [36]	*Design*: randomized controlled trial *Sample*: 24 chronic stroke patients affected by upper limb spasticity *Groups*: BoNT-A + immediate ES vs. BoNT-A + delayed ES *BoNT-A injected*: onabotulinumtoxinA *Injected muscle(s)*: BB, abductor digiti minimi *Total BoNT-A dose injected*: 95 U *Stimulation protocol*: a single 60-min session delivered immediately after injection (immediate ES) followed by 30-min sessions delivered once/day for 3 consecutive days, starting from the day after injection (delayed ES) *Stimulation parameters*: 4 Hz, 0.2 ms, intensity adjusted to elicit muscle contraction *Main findings*: immediate ES leads to greater reduction in spasticity and compound muscle action potential of injected muscles than delayed ES *Side effects reported*: none *PEDro score*: 5
Santamato et al., 2013 [37]	*Design*: randomized controlled trial *Sample*: 32 chronic stroke patients affected by upper limb spasticity *Groups*: BoNT-A + ES vs. BoNT-A + extracorporeal shock-wave therapy *BoNT-A injected*: onabotulinumtoxinA *Injected muscle(s)*: FDS *Total BoNT-A dose injected*: up to 140 U *Stimulation protocol*: 30-min sessions delivered twice/day for 5 consecutive days *Stimulation parameters*: 5 Hz, 50–90 mA *Main findings*: extracorporeal shock-wave therapy lead to greater improvement of spasticity, spasms and pain than ES *Side effects reported*: none *PEDro score*: 6
Baricich et al., 2019 [31]	*Design*: randomized controlled trial *Sample*: 30 chronic stroke patients affected by lower limb spasticity *Groups*: BoNT-A + ES of injected muscle and antagonists vs. BoNT-A + ES of injected muscles only *BoNT-A injected*: onabotulinumtoxinA *Injected muscle(s)*: GM, GL, soleus *Total BoNT-A dose injected*: up to 360 U *Stimulation protocol*: a single 60-min stimulation of injected muscles delivered immediately after BoNT-A injection followed by 60-min sessions delivered to antagonist muscles once/day for 5 consecutive days *Stimulation parameters*: 4 Hz, 0.2 ms *Main findings*: ES of antagonist muscles yields no further clinical benefit *Side effects reported*: none *PEDro score*: 7

BoNT-A, botulinum toxin type A; ES, electrical stimulation; GM, gastrocnemius medialis; GL, gastrocnemius lateralis; TP, tibialis posterior; BB, biceps brachii; Br, brachialis; FCR, flexor carpi radialis; FCU, flexor carpi ulnaris; FDS, flexor digitorum superficialis; FDP, flexor digitorum profundis; PEDro, Physiotherapy Evidence Database.

**Table 2:** Studies on pediatric patients.

Article	Summary
Detrembleur et al., 2002 [38]	*Design*: randomized controlled trial *Sample*: 12 patients with cerebral palsy affected by lower limb spasticity *Groups*: BoNT-A + ES vs. BoNT-A *BoNT-A injected*: onabotulinumtoxinA *Injected muscle(s)*: GM, GL, soleus *Total BoNT-A dose injected*: up to 17.5 U/Kg *Stimulation protocol*: 30-min sessions delivered 6 times/day for 3 consecutive days, starting on the day of BoNT-A injection *Stimulation parameters*: 20 Hz, 0.2 ms, 50–90 mA *Main findings*: combined BoNT-A + ES treatment not superior to BoNT-A *Side effects reported*: none *PEDro score*: 5
Kang et al., 2007 [40]	*Design*: case-control study *Sample*: 18 patients with cerebral palsy affected by lower limb spasticity *Groups*: BoNT-A + ES + rehabilitation vs. BoNT-A + rehabilitation *BoNT-A injected*: onabotulinumtoxinA *Injected muscle(s)*: GM, GL, soleus *Total BoNT-A dose injected*: up to 9.2 U/Kg *Stimulation protocol*: 30-min sessions delivered twice/week for 2 consecutive weeks *Stimulation parameters*: 40 Hz, 0.3 ms, 10–25 mA *Main findings*: ES after BoNT-A injection improved ankle range of motion and gait *Side effects reported*: none *PEDro score*: not applicable (estimated as 5)
Rha et al., 2008 [42]	*Design*: case-control study *Sample*: 23 patients with cerebral palsy affected by lower limb spasticity *Groups*: BoNT-A + high-frequency ES vs. BoNT-A + low-frequency ES vs. sham ES *BoNT-A injected*: onabotulinumtoxinA *Injected muscle(s)*: GM, GL *Total BoNT-A dose injected*: up to 5 U/Kg *Stimulation protocol*: 30-min sessions, delivered once/day for 7 consecutive days *Stimulation parameters*: 4/25 Hz, 0.25 ms *Main findings*: compound muscle action potential reduced by both low- and high-frequency ES, but no significant clinical advantage to ES vs. sham ES *Side effects reported*: none *PEDro score*: not applicable (estimated as 5)
Mudge et al., 2015 [41]	*Design*: randomized controlled trial *Sample*: 6 patients with cerebral palsy affected by lower limb spasticity *Groups*: BoNT-A + ES + stretching vs. BoNT-A + stretching *BoNT-A injected*: onabotulinumtoxinA *Injected muscle(s)*: hamstrings *Total BoNT-A dose injected*: not reported *Stimulation protocol*: 30-min sessions delivered 5 times/week for 12 consecutive weeks *Stimulation parameters*: 50 Hz, 0.26 ms *Main findings*: no difference in passive extensibility of the hamstring *Side effects reported*: none *PEDro score*: 5
Elnaggar et al., 2019 [39]	*Design*: randomized controlled trial *Sample*: 60 patients with cerebral palsy affected by lower limb spasticity *Groups*: ES + rehabilitation vs. BoNT-A + rehabilitation vs. BoNT-A + ES + rehabilitation *BoNT-A injected*: onabotulinumtoxinA *Injected muscle(s)*: GM, GL, soleus *Total BoNT-A dose injected*: up to 12 U/Kg *Stimulation protocol*: 30-min sessions delivered 3 times/week for 12 consecutive weeks *Stimulation parameters*: 30 Hz, 0.25 ms *Main findings*: the integration of ES and BoNT-A influenced ankle biomechanics and postural stability features *Side effects reported*: none *PEDro score*: 6
Yiğitoğlu et al., 2019 [43]	*Design*: randomized controlled trial *Sample*: 40 patients with diplegic cerebral palsy *Groups*: BoNT-A + ES vs. BoNT-A *BoNT-A injected*: onabotulinumtoxinA *Injected muscle(s)*: GM, GL, soleus *Total BoNT-A dose injected*: up to 10 U/Kg *Stimulation protocol*: 20-min sessions delivered 1 time/day for 10 consecutive days *Stimulation parameters*: 40 Hz, 0.35 ms *Main findings*: both groups benefited from the treatment (no additional benefit of ES on BoNT-A was found) *Side effects reported*: none *PEDro score*: 5
Elnaggar et al., 2020 [44]	*Design*: randomized controlled trial *Sample*: 64 patients with cerebral palsy affected by spastic hemiplegia *Groups*: BoNT-A + rehabilitation vs. ES + rehabilitation vs. BoNT-A + ES + rehabilitation vs. rehabilitation *BoNT-A injected*: onabotulinumtoxinA *Injected muscle(s)*: FCU, pronator teres, adductor pollicis, FCR, FPB, brachioradialis, biceps brachii *Total BoNT-A dose injected*: up to 12 U/Kg *Stimulation protocol*: 15-min sessions delivered 1 time/week for 12 consecutive weeks *Stimulation parameters*: 30 Hz, 0.30 ms *Main findings*: patients who received BoNT-A + ES + rehabilitation achieved greater improvement after treatment and a follow-up evaluation *Side effects reported*: none *PEDro score*: not applicable (estimated as 8)

BoNT-A, botulinum toxin type A; ES, electrical stimulation; GM, gastrocnemius medialis; GL, gastrocnemius lateralis; FCU, flexor carpi ulnaris; FCR, flexor carpi radialis; FPB, flexor pollicis brevis; PEDro, Physiotherapy Evidence Database.

## Data Availability

The data presented in this study are available on request from the corresponding author.
